# Coronary Vasospasm After Dobutamine Stress Echocardiogram Triggered by Esmolol

**DOI:** 10.7759/cureus.10015

**Published:** 2020-08-25

**Authors:** Nouraldeen Manasrah, Rohan Naik, Ali F Al Sbihi, Luis C Afonso

**Affiliations:** 1 Internal Medicine, Detroit Medical Center/Sinai Grace Hospital, Detroit, USA; 2 Cardiology, University of Connecticut Health, Farmington, USA; 3 Medicine, Wayne State University, Detroit, USA

**Keywords:** myocardial infarction with no obstructive coronary atherosclerosis, dobutamine stress echocardiography, coronary vasospasm

## Abstract

Dobutamine stress echocardiography (DSE) is a commonly utilized method for coronary artery disease (CAD) diagnosis, and it provides important long-term prognostic information. We report a case of a 53-year-old female with multiple cardiovascular risk factors who underwent DSE for evaluation of underlying CAD. The examination was complicated by wide complex tachycardia and promoted administration of esmolol, which shortly led to ST-segment elevation myocardial infarction (STEMI). Coronary angiography showed complete absence of CAD. Coronary vasospasm was a possible suggested mechanism due to the pharmacologic interaction between beta-blockers and dobutamine on alpha- and beta-adrenergic receptors.

## Introduction

Dobutamine stress echocardiography (DSE) is a commonly utilized non-invasive diagnostic modality in patients with suspected coronary heart disease. Indications of DSE have increased significantly in recent years, leading to more tests and consequently more complications. Although traditionally very safe, several complications of DSE have been reported such as coronary spasm, hypotension, supraventricular or ventricular arrhythmias, and myocardial infarction [1]. We present a patient who developed ST-elevation myocardial infarction (STEMI) during dobutamine stress testing due to coronary vasospasm that was possibly triggered by esmolol. 

## Case presentation

**Figure 1:**
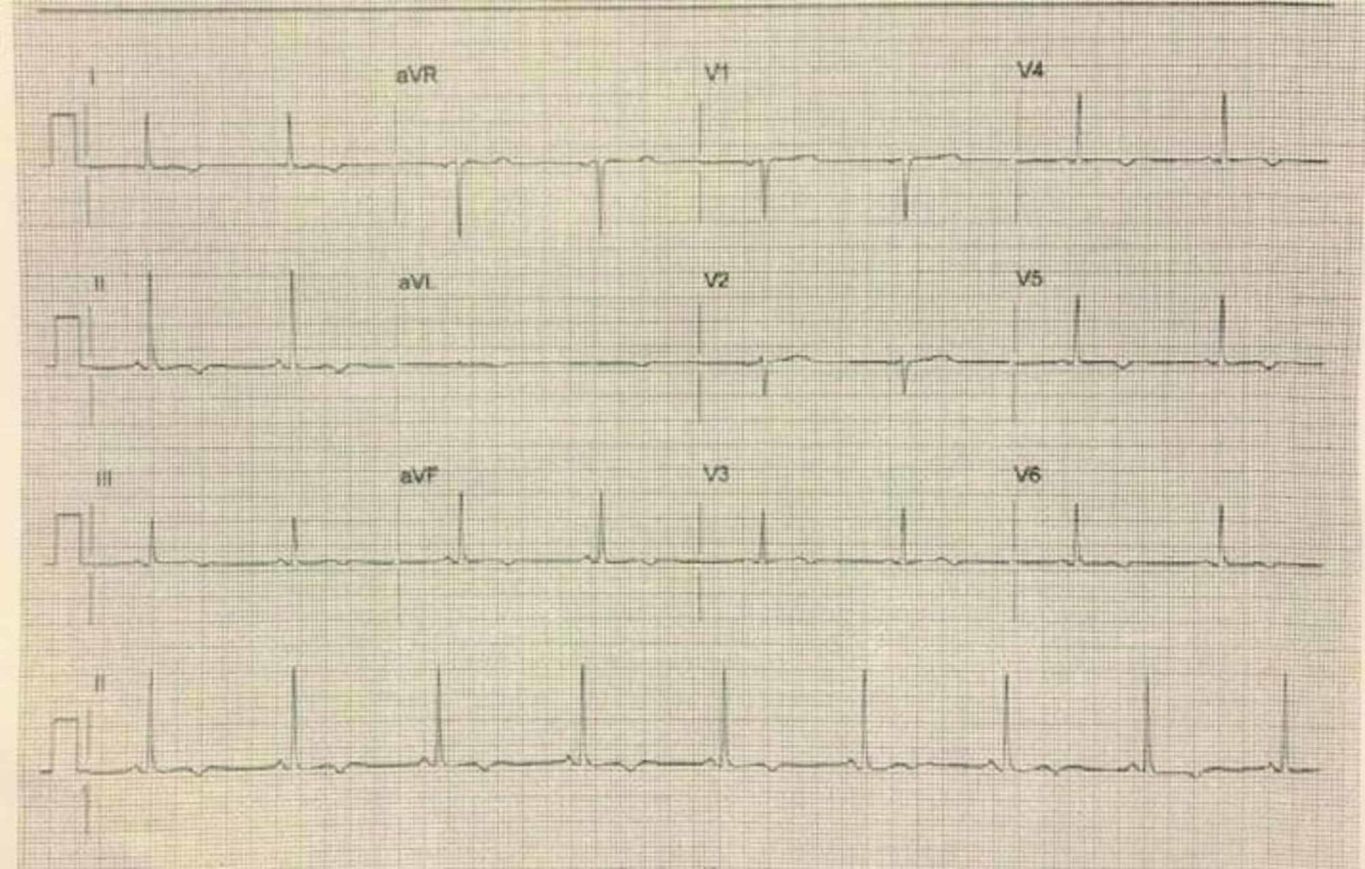
Electrocardiogram (EKG): T-wave inversions in leads V4-V6 and inferior leads II, III, and aVF

A 53-year-old female smoker with a medical history of hypertension and hyperlipidemia presented to our hospital with atypical chest pain of six-hour duration. The pain was insidious in onset, gradually progressive, and was located under her left breast, with radiation to her left arm and back. It was described as burning in quality, reproducible, and non-exertional and improved with ibuprofen. The patient denied any symptoms of shortness of breath, dizziness, diaphoresis, palpitations, nausea, or vomiting. She was able to perform her daily activities without limitations. Physical exam revealed stable vital signs: blood pressure: 123/78 mmHg, heart rate: 73 beats/minute, respiratory rate: 18 breaths/minute, temperature: 36.5 degrees Celsius, SpO_2_: 97% on room air. Cardiovascular examination revealed a pulse with regular rate and rhythm, normal S1 and S2, with no murmurs or added sounds. Respiratory examination was normal with normal vesicular breath sounds heard bilaterally with no adventitious sounds. The rest of physical examination was unremarkable. Electrocardiogram (EKG) on admission was significant for T-wave inversions in leads V4-V6 and inferior leads II, III, and aVF (Figure 1). Serial cardiac troponin-I was negative. All other laboratory evaluation was within normal limits. 

**Figure 2:**
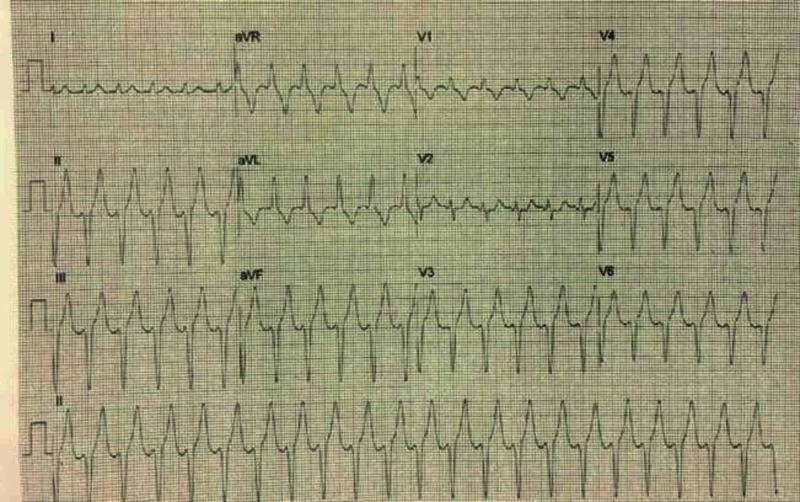
Electrocardiogram (ECG): wide complex tachycardia ECG shows wide complex tachycardia, extreme left axis deviation, R waves in aVR and VI, findings consistent with ventricular tachycardia likely originating in the left ventricle

We used TIMI (Thrombolysis in Myocardial Infarction) risk score [2] and HEART (History, ECG, Age, Risk factors and Troponin) score [3], which are useful tools for early risk stratification; they help in making decisions about patient's management and predict the likelihood of adverse cardiac events. The calculated scores were 2 and 5, respectively, so our patient underwent DSE to evaluate for inducible ischemia and underlying coronary artery disease (CAD). Intravenous dobutamine infusion was started at 10 mcg/kg/min, increasing to 50 mcg/kg/min in three-minute stages. Intravenous atropine (0.75 mg) was also administered, so 85% of maximum predicted heart rate could be achieved. Subsequently, continuous EKG showed significant tachycardia 170 beats/minute, along with QRS widening (Figure 2).

**Figure 3:**
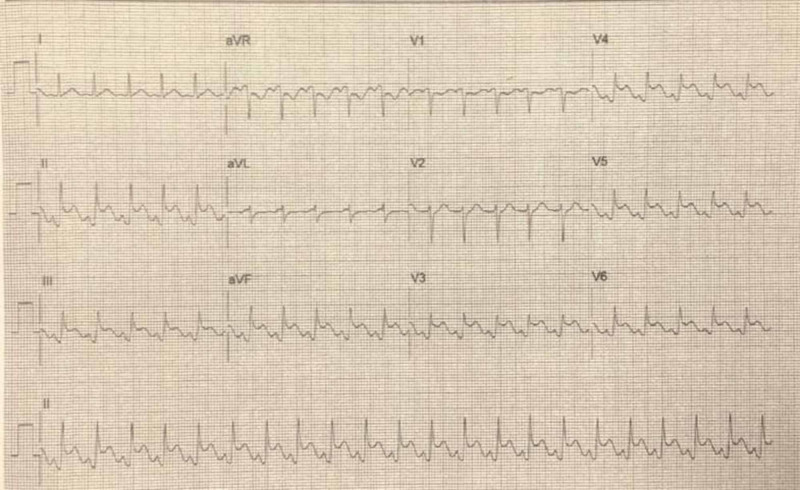
Electrocardiogram (ECG): ST-segment elevations in inferior and anterolateral leads II, III, aVF, and V3-V6 Continuous EKG showed ischemic ST changes with J-point elevation, PR depression, and 3 mm ST-segment elevations in inferior and anterolateral leads II, III, aVF, and V3-V6

This prompted administration of intravenous esmolol 15 mg and dobutamine infusion was stopped, and QRS duration reverted back to the baseline. Shortly thereafter, the patient complained of substernal chest pain that was different from the chest pain that brought her to the hospital. It was sudden, rapidly progressive, substernal in location, and described as tightness and heaviness. Continuous EKG showed ST-segment elevations in inferior and anterolateral leads II, III, aVF, and V3-V6 (Figure 3).

Sublingual nitroglycerin was given immediately in an attempt to relieve the substernal chest pain. Loading doses of aspirin and ticagrelor were administered. Cardiac troponins were drawn, and the patient was taken for emergency left heart catheterization (LHC). Contrary to our expectation, LHC showed complete absence of CAD. Cardiac troponin-I was elevated at 1.29 ng/ml. Subsequent troponin drawn three hours later showed a drop down to 0.41 ng/ml. The chest pain and ST-segment elevations persisted for about 20 minutes with gradual resolution following administration of sublingual nitroglycerin. Subsequent 12-lead EKG was normal. Resting echocardiogram showed left ventricular ejection fraction of 67%, no new regional wall motion abnormalities, and no valvular abnormalities. Echocardiographic response to stress did not reveal any new regional wall motion abnormalities, but it did show dilation of the left ventricular cavity in recovery.

The patient was monitored in cardiac telemetry unit for two days, counseled on smoking cessation, and discharged on her home medications of amlodipine, hydralazine, and atorvastatin. In aggregate, findings were deemed secondary to coronary vasospasm in the mid-left anterior descending (wrap around) distribution and possibly also in the right coronary artery distribution.

## Discussion

Dobutamine is a synthetic catecholamine with a relatively short plasma half-life; it has strong β1-receptor, moderate β2-receptor, and mild α1-receptor agonist activity (5+, 3+, and 1+ potency, respectively). In DSE, it is used at high doses (20-40 mg/kg/min) which induce an increase in myocardial oxygen demand and in the setting of flow limiting stenosis; it reveals wall motion abnormalities indicating the presence of underlying CAD. Data from 26 studies in a meta-analysis reported the incidence of potentially life-threatening complications associated with DSE is <0.01% [4]. Despite this relatively good safety profile, approximately half of patients experience some reaction to the dobutamine infusion, including nausea, flushing, headache, neck/chest pounding, paresthesia, urinary urgency, palpitations, or dyspnea. Other less frequent complications may occur such as myocardial infarction, hypotension, and coronary artery spasm. The estimated prevalence of coronary artery spasm during DSE is 0.4% [5]. The conspicuous absence of CAD in this female patient with cardiovascular risk factors who developed typical chest pain and diffuse ST elevations during DSE presented a diagnostic dilemma.

Our differential diagnosis at that time included coronary artery vasospasm, Takotsubo cardiomyopathy, and spontaneous coronary artery dissection (SCAD), among others. The absence of typical echocardiographic findings associated with Takotsubo cardiomyopathy and the lack of typical emotional stressor made this diagnosis less likely. However, there have been reports of dobutamine itself being responsible for Takotsubo cardiomyopathy [6]. Another consideration was an SCAD, and our patient was relatively young female with risk factors for coronary dissection. SCAD, especially type 2 and type 3, can be a challenging diagnosis to make purely angiographically without intracoronary imaging, and requires a high index of suspicion to diagnose accurately. However, the complete resolution of angina symptoms and electrocardiographic abnormalities following administration of sublingual nitroglycerin clearly supports a diagnosis of dobutamine-esmolol-induced coronary vasospasm. Female sex, smoking, dyslipidemia, and hypertension are all risk factors for coronary spasm.

Although the exact etiology of the vasospasm is unclear, one possible mechanism is the pharmacologic interaction between beta-blockers and dobutamine on alpha- and beta-adrenergic receptors. There is significant overlap in receptor activity, with a possible imbalance created by the mild alpha-1 vasoconstrictor effect of dobutamine and its moderate beta-2 receptor vasodilator effect. Administration of a beta-blocker can cause further imbalance. The resulting vasospasm was significant enough to cause a rise in cardiac troponin which had been undetectable prior to stress testing. Esmolol is effective and well tolerated for the management of dobutamine-related tachycardia, but in our case, it induced coronary vasospasm. A careful review of literature identified two similar case reports of vasospasm during DSE induced by esmolol [7,8].

Endothelial dysfunction could also be a key factor in the pathogenesis of the dobutamine-induced vasospasm [9]. In the presence of endothelial dysfunction, it seems that the vasodilator predominance of dobutamine on β2 receptor is lost in favor of a vasoconstrictor response on ά1 receptor. Moreover, in patients with endothelial dysfunction, endothelium-derived nitric oxide activity is deficient, making them hyper-responsive to precipitating factors for coronary spasm, including dobutamine that is not known to do so in normal endothelium [10].

## Conclusions

Our illustrative case highlights a rare complication of DSE and serves to alert the clinicians on the differential, mechanisms, and clinical approach to managing ST-elevation during the course of pharmacologic stress echocardiography. Coronary vasodilators like nitroglycerin are usually effective in treating coronary vasospasm. This case also highlights the fact that dobutamine-induced vasospasm may mimic acute coronary syndrome and often requires cardiac catheterization to exclude thrombosis as a pathological cause of STEMI. In the future, if stress testing is needed in such patients, vasodilator stress testing should be preferred because this patient has displayed sensitivity to catecholamines and will likely remain at increased risk for coronary spasm. Among young individuals presenting with a STEMI, non-atherosclerotic causes of MI like spasm, embolism, dissection, and vasculitis must be considered.
